# Solution structures and biophysical analysis of full-length group A PAKs reveal they are monomeric and auto-inhibited in *cis*

**DOI:** 10.1042/BCJ20180867

**Published:** 2019-04-04

**Authors:** Fiona J. Sorrell, Lena Marie Kilian, Jonathan M. Elkins

**Affiliations:** 1 Structural Genomics Consortium, University of Oxford, Old Road Campus Research Building, Roosevelt Drive, Oxford OX3 7DQ, U.K.; 2 Structural Genomics Consortium, Universidade Estadual de Campinas, Cidade Universitária Zeferino Vaz, Av. Dr. André Tosello, 550, Barão Geraldo, Campinas SP 13083-886, Brazil

**Keywords:** kinase, SAXS, structure

## Abstract

The group A p21-activated kinases (PAKs) exist in an auto-inhibited form until activated by GTPase binding and auto-phosphorylation. In the auto-inhibited form, a regulatory domain binds to the kinase domain (KD) blocking the binding of substrates, and CDC42 or Rac binding to the regulatory domain relieves this auto-inhibition allowing auto-phosphorylation on the KD activation loop. We have determined the crystal structure of the PAK3 catalytic domain and by small angle X-ray scattering, the solution-phase structures of full-length inactive PAK1 and PAK3. The structures reveal a compact but elongated molecular shape that demonstrates that, together with multiple independent biophysical measurements and in contrast with previous assumptions, group A PAKs are monomeric both before and after activation, consistent with an activation mechanism of *cis*-auto-inhibition and initial *cis*-auto-phosphorylation, followed by transient dimerisation to allow *trans*-auto-phosphorylation for full activation, yielding a monomeric active PAK protein.

## Introduction

There are six human p21-activated kinases (PAKs) subdivided into two groups based on functional and sequence similarity. PAK1, PAK2 and PAK3 are the group A or group 1 PAKs, whereas PAK4, PAK5 and PAK6 are group B or group 2 [1]. PAKs act as effector proteins for the Rho GTPases CDC42 and Rac and transmit signals for the regulation of the cytoskeleton, growth, development, proliferation and survival [2–4]. PAKs, and in particular PAK1, have been of great interest as anti-cancer targets and potentially for other disorders such as Down syndrome [5] and fragile X syndrome [6]. However, the interest in PAK inhibition is tempered by the cardiotoxicity shown by pan-group A PAK inhibitors [7]; PAK2 may be the critical gene as while PAK1 knockout mice are viable, PAK2 knockout mice are not. Interestingly, down-regulation or deficit of function of group A PAKs has also been linked to various diseases, especially neurodegenerative disorders [8,9] including Alzheimer's disease [10], Parkinson's disease [11], non-syndromic X-linked mental retardation [12,13] and intellectual disability disorders [14]. PAK1 deficiency leads to cardiac hypertrophy and increased risk of mortality related to heart failure, and PAK1 or PAK2 activation may be beneficial in hypertrophy and heart failure [15–20].

Group A PAKs have a GTPase-binding domain (GBD, also known as CDC42/Rac interactive binding (CRIB) domain) N-terminal to an auto-inhibitory domain (AID) and a serine/threonine protein kinase domain (KD) that is highly conserved (Supplementary Figure S1). In the un-activated state, the AID sterically blocks the substrate-binding site, preventing both the phosphorylation of the activation loop that is required for full activation of the KD and the phosphorylation of other substrates [21,22]. For activation of PAK, GTP-bound CDC42 or Rac binds to the GBD which relieves the auto-inhibition, allowing activation loop phosphorylation and then substrate phosphorylation. One of the proposed differences between group A and group B PAKs is that group A PAKs are dimeric before activation, whereas group B PAKs are monomeric [2,4,23]. Group A PAKs have been considered dimeric since the first crystal structure of PAK1 was obtained [22]. This structure consisted of the regulatory domain of PAK1 (residues 70–149) in complex with an inactive mutant (K299R) of the KD (residues 249–545) and revealed an unusual asymmetric dimeric interaction between two strands of the regulatory domain [22]. It was suggested that the removal of the AID was coupled with disruption of the *trans* dimer so that auto-phosphorylation occurs in *cis* on monomeric PAK [21,22]. Later evidence suggested that PAK2 undergoes intermolecular auto-phosphorylation and, furthermore, the catalytic domain of phosphorylated PAK2 was found to dimerise in solution [24,25]. Subsequent careful investigation of the mechanism using enzyme kinetics revealed a two-step mechanism whereby upon release of the AID through CDC42/Rac-binding PAK2 is initially activated in *cis*, followed by propagation in *trans* [26].

Group B PAKs have been relatively well-studied structurally, including the full-length (FL) PAK4 both alone (PDB 4fie) [27] and in complex with CDC42 (PDB 5upk, 5upl) [28], and the catalytic domains of PAK4, PAK5 and PAK6 in complex with interaction partners, peptides and inhibitors (e.g., PDB 2c30, 2f57, 4fif, 4l67, 4xbu, 5i0b, 5vee, 5xvg) [27,29–34]. Aside from the PAK1 KD–AID complex mentioned above (PDB 1f3m) [22], structural information for the group A PAKs is predominantly limited to the PAK1 catalytic domain (encompassing residues 248–545), including complexes with various nucleotides and ligands (e.g., PDB 3q4z, 3q53, 3fy0, 4daw, 4eqc, 4zlo, 5bkq, 5dfp, etc.) [35–40]. Typically, the K299R mutant thought to produce catalytically inactive ‘kinase-dead’ protein, has been used for crystallisation since the production of the wild-type catalytic domain results in the death of the host organism during overexpression [21,22]. This single mutation was later found to retain basal activity towards substrates and was even found to be fully auto-phosphorylated during expression and purification, with a phosphorylated T423 in the kinase activation loop visible in the crystal structure (PDB 3q52) [35]. A double mutant (K229R/D389N) (PDB 3q4z) was required to generate inactive, unphosphorylated PAK1 [35]. The structures have proved to be useful in the study of the PAK1 activation mechanism and in the development of small molecule inhibitors [7,22,35–42]. For PAK2, structural information consists only of a handful of crystal structures containing short (16–20 mer) peptide fragments from the PAK2 N-terminus in complex with potential interaction partners (PDB 3pcs, 2df6, 2g6f) [43,44], and PAK3 has been altogether neglected.

With the dual aims of determining the structures of the FL group A PAKs and analysing the opportunities for allosteric inhibitor or activator development, we generated purified samples of FL wild-type PAK1 and PAK3, determined the solution-phase structures, and generated the first crystal structure of the PAK3 catalytic domain. The structures of inactive and auto-phosphorylated PAK1 and PAK3 reveal for the first time the overall size and shape of the FL proteins and hint at possibilities for their domain organisation. Most surprisingly, together with a comprehensive biophysical analysis, they show that they are, contrary to previous evidence, monomeric in solution both in their inactive state and following auto-phosphorylation. Based on these data and the known kinetic parameters of PAK2 activation, we propose an updated model for group A PAK auto-inhibition.

## Materials and methods

### Recombinant protein expression and purification

PAK and CDC42 constructs were cloned into expression vector pNIC28-Bsa4 which adds an N-terminal hexahistidine tag and a TEV protease recognition site for removal of the tag [45]. The constructs were transformed into *Escherichia coli* strain BL21(DE3)-R3 along with a plasmid expressing the bacteriophage lambda phosphatase and three rare tRNAs, to ensure production of the unphosphorylated kinases. Cells were grown at 37°C in LB medium (Miller) to an OD_600_ of 0.4–0.5 and temperature lowered to 18°C prior to induction of protein expression by the addition of 0.4 mM isopropyl 1-thio-d-galactopyranoside at an OD_600_ of 0.6. The following morning cells were harvested by centrifugation and resuspended in lysis buffer (50 mM HEPES (pH 7.5), 500 mM NaCl, 5% glycerol, 20 mM imidazole, 0.5 mM TCEP, 1:1000 dilution of Merck protease inhibitor cocktail set III). Pellets were stored frozen until required, then gently thawed in lukewarm water and lysed by sonication on ice. Proteins were purified by immobilised metal affinity chromatography (IMAC) using Ni-Sepharose resin (GE Healthcare) in a gravity flow column and eluted by addition of a buffer containing 100–250 mM imidazole. Removal of the hexahistidine tag was carried out by addition of recombinant TEV protease during overnight dialysis into a buffer without imidazole, followed by purification on a second IMAC column and finally by size-exclusion chromatography (SEC) (Superdex 75 or Superdex 200 16/600, GE Healthcare) in a buffer consisting of 50 mM HEPES (pH 7.5), 300–500 mM NaCl, 5% glycerol, 0.5 mM TCEP, 1:1000 dilution of Merck protease inhibitor cocktail set III. For CDC42, all purification buffers contained an additional 50 µM GDP and 5 mM magnesium chloride.

To prepare activated CDC42, the nucleotide was exchanged by addition of 10 mM EDTA, 1 mM DTT, 100 U of calf intestinal alkaline phosphatase (CIP) and 0.55 mM GppCp (β,γ-methyleneguanosine 5′-triphosphate) with overnight incubation at 4°C, followed by SEC, as before, and substituting 50 µM GppCp for GDP in the SEC buffer.

For PAK KD constructs, a final purification step of ion exchange chromatography was required to separate a small amount of phosphorylated protein from the unphosphorylated material. PAK KD constructs in a buffer containing ∼15 mM NaCl were loaded onto a 5 ml HiTrap Q column in low salt buffer (50 mM HEPES (pH 7.5), 5% glycerol, 0.5 mM TCEP) and eluted using a gradient of 0–100% high salt buffer (1 M NaCl, 50 mM HEPES (pH 7.5), 5% glycerol, 0.5 mM TCEP).

Purified proteins were characterised using denaturing liquid chromatography–mass spectrometry (LC–MS) and SDS–PAGE, then flash frozen in liquid nitrogen and stored at −80°C until required.

### *In vitro* protein phosphorylation

Purified, unphosphorylated, PAK1 and PAK3 constructs were auto-phosphorylated by addition of 1:100 molar ratio of CDC42:GppCp, 5 mM ATP and 5 mM magnesium chloride for several hours at 4°C in a reaction buffer consisting of 50 mM HEPES (pH 7.4), 0.02% NaN_3_, 0.01% BSA, 0.1 mM sodium orthovanadate. Phosphorylation state was characterised by denaturing mass spectrometry using an electrospray ionisation (ESI) time-of-flight (LC/MSD TOF) spectrometer. Excess nucleotide was removed and phosphorylated protein was buffer exchanged into 50 mM HEPES (pH 7.4), 150 mM NaCl by SEC using a Superdex 75 or Superdex 200 16/600 column, as mentioned before. Proteins were concentrated using a spin filter centrifugal device with 30 kDa molecular mass cut-off.

### Analytical size-exclusion chromatography

Purified PAK constructs were concentrated to 2–8 mg/ml and centrifuged at 21,000×*g* for 10 min to remove aggregates. About 15 µl of sample was injected onto a Superdex 200 5/150 GL analytical SEC column (GE Healthcare) pre-equilibrated at 4°C in a buffer consisting of 50 mM HEPES (pH 7.5), 200 mM NaCl, 5% glycerol, 0.5 mM TCEP. Detection was by UV absorbance at 280 nm and the eluted protein retention volume was determined based on the UV_280_ peak maximum. Commercial molecular mass standards (Bio-Rad Catalogue #151-1901) as well as an in-house molecular mass marker (a KD construct of known molecular mass) were periodically injected onto the column to ensure constant calibration. Two methods were used for the estimation of molecular mass. Calibration curves were constructed based on the retention volume of the molecular mass standards run in triplicate and plotted against either the log of the molecular mass or plotted against literature values obtained for the Stokes radius (*R*_s_). Linear regression in GraphPad Prism 7 software was used to determine the values of the unknowns from their retention time. An average estimate of the molecular mass of each PAK sample was determined based on the retention volume of three separate SEC runs compared with the calibration curve. For determination of molecular mass using the Svedberg equation, $M\;=\;SN_{0}(6\pi \eta R_{s})/(1-\nu 2\rho )$, we followed the method described by Erickson [46], assuming *η* = 0.01, *ν*2*ρ* = 0.73. We used the sedimentation coefficient determined by analytical ultracentrifugation–sedimentation velocity (AUC-SV), extrapolated to standard conditions using SEDNTERP and combined with the *R*_s_ value determined experimentally.

### Analytical ultracentrifugation

AUC-SV was performed at 4°C using purified proteins that had been dialysed into AUC buffer (50 mM HEPES (pH 7.5), 150 mM NaCl) overnight. The dialysis buffer was used in the reference cell. Protein concentrations were 0.5–1.0 mg/ml. Data were measured at 40,000 rpm using absorbance optics in a Beckman XL-I Analytical Ultracentrifuge equipped with a Ti-50 rotor. Two-sector cells fitted with either carbon-epon or aluminium centrepieces and with sapphire windows were filled with sample and AUC buffer as a reference. Data were analysed using SEDFIT [47] to calculate c(*s*) distributions. SEDNTERP was used to calculate the protein partial specific volume, buffer density and viscosity. The maximum possible sedimentation coefficient (*s*_max_), corresponding to an unhydrated smooth sphere of mass equal to the protein under investigation, was calculated as described [46] and the ratio of *s*_max_/*s* was then determined.

Analytical ultracentrifugation–sedimentation equilibrium (AUC-SE) experiments were performed at 4°C in AUC buffer using six-sector carbon-epon centrepieces and three protein loading concentrations, and AUC buffer as a reference. Three sequentially increasing rotor speeds were used (see Figure 2c,d) over 96 h and equilibrium was monitored using absorbance at 279 nm. SEDPHAT [48] was used to perform a global fit of the data to a ‘single species of an interacting system’ model in order to determine the apparent molar mass and extinction coefficient. Mass conservation was switched on, baseline fitting performed with bottom allowed to float, and TI (time-invariant) and RI (radial-invariant) noise was subtracted from the raw data.

### Liquid chromatography–mass spectrometry

For denaturing LC–MS, 1 µl protein at 1 mg/ml was denatured by addition of 59 µl of 0.1% formic acid and loaded onto an ESI-TOF (LC/MSD TOF) spectrometer. For non-denaturing MS, proteins were buffer exchanged using Micro Bio-Spin 6 size exclusion columns (Bio-Rad) into 50 mM ammonium acetate (pH 6.5) prior to loading directly on an ESI-TOF spectrometer.

### High-performance liquid chromatography–small angle X-ray scattering (HPLC-SAXS)

Full experiment details of SAXS measurements are shown in Supplementary Table S1 (PAK1) and Supplementary Table S2 (PAK3). Recombinant unphosphorylated or auto-phosphorylated PAK proteins were purified as described and checked for monodispersity using AUC and SEC. All samples were concentrated into the range of 5–32 mg/ml and flash frozen in liquid nitrogen. The thawed samples were injected onto a 4.6 ml Shodex 403 silica SEC column and HPLC-SAXS data were collected at 15°C and a flow rate of 0.16 ml/min with continuous 1 s data-frame measurement of SEC elution using Diamond beamline b21 equipped with a Dectris Pilatus 2M detector and camera length of 4.014 m, at a wavelength of 1 Å and *q* measurement range of 0.0022–0.42 Å^−1^. Running buffer consisted of 25 mM HEPES (pH 7.5), 150–200 mM NaCl, 0.5 mM TCEP, 2–2.5% glycerol, 1% sucrose. Data were processed and analysed using ScÅtter 3.0 software [49]. Buffer subtraction was performed separately for a range of stable *R*_g_ values across each intensity peak using the SEC flow-through prior to elution of the protein. The radius of gyration (*R*_g_) was determined using both Guinier fitting and by analysis of the *P*(*r*) distribution. Maximum particle dimension (*d*_max_) and volume of correlation (*V*_c_) were also determined. The molecular mass of the proteins was determined using the *Q*_R_ method [50]. A series of models for the FL and ΔN-truncated PAK proteins were generated using MODELLER [51] and/or I-TASSER [52]. For PAK1 ΔN, the crystal structure of the PAK1 KD (residues 249–545) complexed with the auto-inhibitory fragment (residues 70–149), PDB 1f3m Chains A and C, was used as a template and the 100 missing residues from the segment connecting the kinase and inhibitory fragments were modelled in various conformations. For the PAK3 ΔN sample, the crystal structure of the PAK3 KD was used as a template for this region of the protein. This process was repeated for the FL proteins. FoXS [53] was used to simulate scattering curves from the generated models and compare them to the experimental data. Ambimeter [54] was used to quantitatively assess the shape reconstruction prior to *ab initio* modelling. The reduced experimental SAXS data were then input into Gasbor [55] and a dummy residue model generated. SUPCOMB [56] was then run to superpose the final models against a relevant crystal structure, where available. Pymol was used to generate figures.

### Structure determination of PAK3 kinase domain

The purified unphosphorylated KD construct of PAK3_A280D_ (residues 246–544 in isoform A), was concentrated to 11 mg/ml and mixed with 5 mM ATP-γ-S and 2 mM MgCl_2_ and incubated on ice for a minimum of 30 min. The sample was centrifuged at 15,860×*g*, 4°C for 15 min immediately prior to setting up crystallisation plates. Crystallisation sitting-drop vapour diffusion experiments were set up by Mosquito robot (TTP Labtech) using protein to reservoir ratios of 2:1, 1:1 and 1:2 in 150 nL total volume and equilibrated against 20 µl of reservoir solution. Crystals grew from several different conditions within 24 h and were harvested using reservoir solution supplemented with 25% ethylene glycol as a cryo-protectant prior to flash freezing in liquid nitrogen. X-ray data were collected at Diamond Light Source beamline I03 and processed using XIA2 [57]. Molecular replacement was carried out in Phaser [58] using PAK1 structure (PDB code 3q4z: chain A) as a search model. Refinement was carried out using PHENIX [59]. MolProbity [60] and JCSG check (Joint Center for Structural Genomics) was used to validate the model prior to deposition in the PDB under accession code 6fd3. Processing and refinement statistics are given in Supplementary Table S3.

## Results

### Full-length group A PAK proteins can be produced in their un-activated state

**Figure 1.**
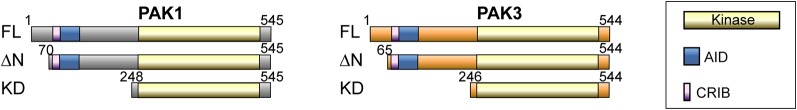
PAK experimental constructs and domain boundaries. FL PAK1 and PAK3 as well as two truncated proteins were produced. The truncated proteins represent either the KD alone (KD) or an N-terminally truncated construct (ΔN) similar to that previously used by Lei et al. [22]. Abbreviations: CRIB, CDC42/Rac interactive binding domain; AID, auto-inhibitory domain.

To test whether the structure or oligomerisation are influenced by the presence of the protein N-terminus or the AID or GBD domains, three different constructs were prepared for each of PAK1 and PAK3: the FL protein, the KD alone, or a construct lacking the N-terminal 69 residues (ΔN), matching the PAK1 protein used by Lei et al. [22] (Figure 1). It was previously reported that recombinant expression of active group A PAKs in *E. coli* led to death of the micro-organism and low expression yield [21,22] and consequently many structural and mechanistic studies have used protein incorporating the K299R mutation, thought to be inactive, but which was later discovered to retain basal catalytic activity [35]. We overcame this problem by co-expression of the wild-type proteins with a phosphatase to produce the proteins in an unphosphorylated, un-activated, state, confirmed by LC–MS (Supplementary Figure S2). The longer constructs were completely unphosphorylated after purification, but the isolated KD constructs contained a small amount of phosphorylated protein, despite the phosphatase co-expression, and required a subsequent purification step to separate the unphosphorylated and phosphorylated material (Supplementary Figure S2).

### Group A PAKs are monomeric in solution before activation

Analytical SEC of the FL, unphosphorylated proteins resulted in an elution profile consisting of one major peak (>∼85% of the sample) (Supplementary Figure S3a,b). A molecular mass estimate for the proteins was determined by comparing their SEC elution volume against a calibration curve (Supplementary Figure S3c), as had been performed previously for PAK1 [21,22,61]. These data were inconclusive and masses between monomeric and dimeric forms were obtained (Supplementary Figure S3e). Since SEC elution volume is proportional to the molecular size and surface properties rather than the molecular mass of the particle, elution volume can be strongly influenced by factors such as particle shape, sphere of hydration and frictional drag. We, therefore, sought to obtain more accurate estimates of the molecular mass of the principal species using alternative methods.

**Figure 2.**
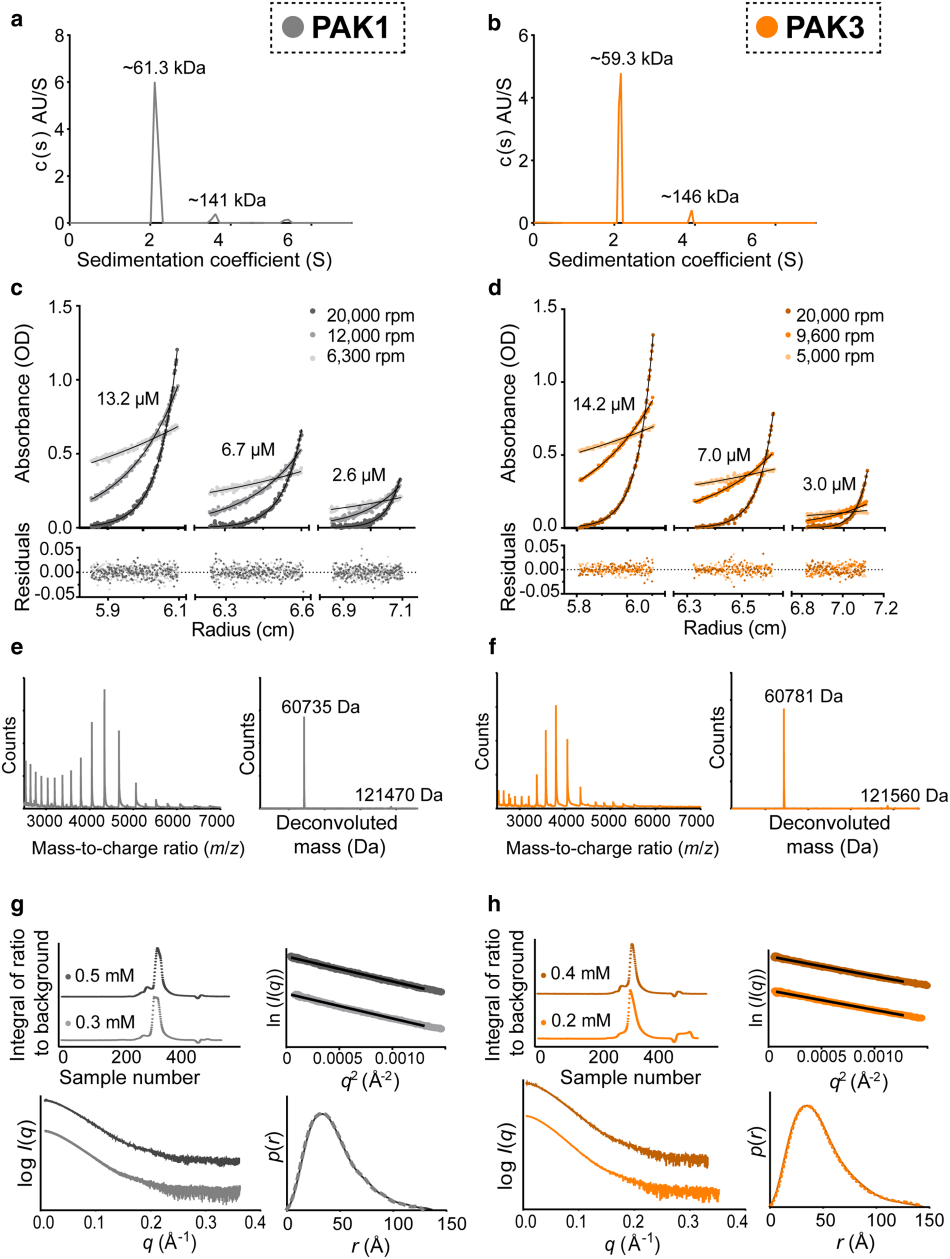
FL PAK1 (grey) and PAK3 (orange) are monomeric in solution in their un-activated state. (**a**,**b**) Representative AUC-SV measurements of PAK1 and PAK3 with sedimentation coefficients converted to molecular mass for each peak in the c(s) plot. (**c**,**d**) AUC-SE measurements for PAK1 and PAK3 at three different concentrations and rotor speeds. Black lines indicate global fitting of raw data to a single species model. (**e**,**f**) Native-MS spectra (left) and corresponding deconvoluted spectra (right) of PAK1 and PAK3. (**g**) Two independent HPLC-SAXS measurements of PAK1 were made: (top left) normalised HPLC-SAXS elution trace with a concentration of the injected sample indicated and (bottom left) corresponding buffer-subtracted SAXS *q* versus log *I*(*q*) scattering profile for the major peak. (top right) Guinier/reciprocal space analysis of both samples and (bottom right) normalised *p*(*r*) distribution for determination of *R*_g_ and *I*(0) (Supplementary Tables S1 and S2), both used to estimate molecular mass by the *Q*_R_ method. (**h**) Corresponding HPLC-SAXS elution profile, scattering profile, Guinier and real-space plots for PAK3.

AUC-SV of PAK FL proteins resulted in sedimentation coefficients that were unexpectedly consistent with monomeric species (Figure 2a,b and Table 1). For example, FL PAK1 has a calculated molecular mass of 60.7 kDa, confirmed experimentally by denaturing LC–MS (Supplementary Figure S2), and had a sedimentation coefficient consistent with a particle of mass 61.3 kDa by AUC-SV (Figure 2a), within 1% of the expected value for a monomer. Smaller peaks (<5% of total amount of material) corresponding to higher molecular mass species could be observed, which might perhaps be evidence for small amounts of dimer formation, but may alternatively represent experimental artefacts. For example, for PAK3 a secondary peak corresponding to 4.8% of material had an S*_w_* of 3.9, corresponding to a molecular mass of ∼146 kDa (Figure 2b).

**Table 1:** Summary of molecular sizes determined by different techniques, showing monomeric PAK proteins in all cases All reported masses are for the major species present, in units of kilodaltons (kDa), according to the procedures detailed in experimental methods. *M*_CALC_ = molecular mass calculated based on the amino acid sequence of protein construct. For experimental methods, see text. For SAXS data, the value is determined using *Q*_R_ method reciprocal space estimates for *I*(0) and *R*_g_, with masses determined from real-space values reported in parentheses. Duplicate independent measurements were made for AUC-SV and for FL PAKs in SAXS and average values reported.

			Unphosphorylated						Phosphorylated	
	Construct	*M*_CALC_	*M*_LC–MS_	*M*_SEC_	*M*_AUC-SV_	*M*_AUC-SE_	*M*_N-MS_	*M*_SAXS_	*M*_AUC_	*M*_SAXS_
PAK1	FL	60.7	60.7	61.7	63.9	61.3	60.7	64 (61)	57.3	63 (57)
	ΔN	53.3	53.3	56.6	54.3			54 (55)		
	KD	33.4	33.4	35.2				32 (42)		32 (40)
PAK3	FL	60.8	60.8	66	57.1	55.8	60.8	69 (67)		69 (69)
	ΔN	53.9	53.9	48	53.8			55 (63)		
	KD	33.6	33.6							34 (40)

As previous reports suggested that the PAKs are dimeric, to gain accurate estimates of the apparent molecular masses independent of their shape, we used AUC-SE (Figure 2c,d). Global fitting of the data to a single species model (black line, Figure 2c,d) led to local root mean square deviations less than 0.01 in all cases and a random and small the distribution of residuals (bottom panel, Figure 2c,d), indicating an acceptable fit of the model to the data. The resulting apparent molar masses again closely matched monomeric species (Table 1). Following AUC analysis, each sample was analysed by SDS–PAGE and/or LC–MS to ensure it had not undergone degradation during the course of the experiment. Using the Svedberg equation to calculate the molecular mass from the Stokes radius (*R*_s_; defined as the radius of a smooth sphere with the frictional coefficient equal to the protein under investigation) obtained from SEC with the sedimentation coefficient from AUC [46] gave molecular masses within 10% of that expected for monomeric protein (Table 1 and Supplementary Figure S3d,e). This emphasises that the PAK FL proteins have substantially non-ideal behaviour on SEC.

ESI mass spectrometry under non-denaturing conditions (‘native-MS’) demonstrated intact monomeric PAK FL proteins (Figure 2e,f), with a small amount of unfolded monomer also evident at low mass-to-charge ratios in the native-MS spectra, most likely caused by desalting and exchange of the buffer into conditions compatible with MS. There were minor peaks for dimeric PAK FL proteins, however, with ESI mass spectrometry artefactual higher-order oligomers are commonly observed in low proportions [62,63] and so the presence of minor peaks for dimeric PAK FL proteins is not of itself evidence of the ability to oligomerise.

Finally, the PAK1 FL and PAK3 FL proteins were analysed using HPLC-SAXS in two independent measurements. To test the possibility that there was a monomer–dimer equilibrium we used extremely high protein concentrations. PAK1 was injected onto the SEC column at loading concentrations of 15 and 32 mg/ml (0.25 and 0.52 mM), and PAK3 was tested at 11 and 22 mg/ml (0.18 and 0.36 mM). In each case, a major peak was eluted consisting of >∼95% of the sample material (Figure 2g,h). The SAXS scattering data were analysed to determine the radius of gyration (*R*_g_), forward scattering (*I*(0)) and volume of correlation (*V*_c_) using both Guinier and real-space plots (Figure 2g,h) to allow calculation of the molecular mass of the particles in solution using the *Q*_R_ method, which can determine particle mass without making assumptions about the concentration or shape of the particles [50]. Once again the unphosphorylated PAK FL proteins were monomeric (Table 1). A minor peak or shoulder was visible in the samples with weak scattering intensity which could indicate a very small population of higher order oligomers, however, the calculated *R*_g_ was not consistent across this peak indicating that a mixture of species was present.

Taken together, all the data indicated that PAK FL proteins were monomeric, and no definitive evidence for the presence of dimeric PAK FL was observed, even at very high protein concentrations. Since some previous reports of dimeric group A PAKs were based on N-terminally truncated constructs [21,22], we tested whether the absence of the N-terminal 69 residues was important for oligomerisation. The PAK ΔN constructs were assessed using AUC-SV, SAXS and SEC as for the PAK FL constructs and were also monomeric in solution (Table 1), with the proviso that in AUC-SV there was again some evidence of higher molecular mass material (<5% of the total sample) which could be attributed to either dimer formation or experimental artefact. By SEC, we observed that the ΔN proteins exhibited non-ideal behaviour on the column, similar to FL proteins (Supplementary Figure S3). We also tested the isolated KD of PAK1 in SEC and SAXS experiments, which was found to be monomeric. It, therefore, appears that monomers are the major state of inactive group A PAK proteins.

### Group A PAKs remain monomeric in solution after activation

**Figure 3.**
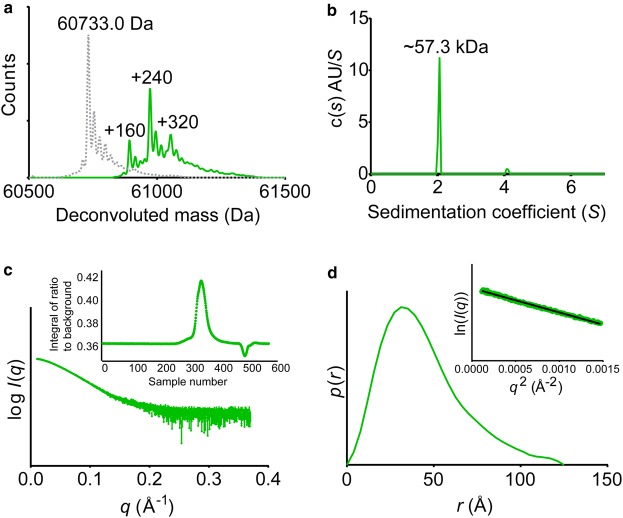
FL PAK1 remains monomeric after activation. (**a**) Overlaid denaturing LC–MS spectra of PAK1 FL prior to (grey dotted line) and following (green line) auto-phosphorylation. (**b**) AUC-SV analysis of phosphorylated PAK1 FL. (**c**) SAXS scattering profile of phospho-PAK1 FL generated from the buffer-subtracted major peak of the HPLC-SAXS profile (shown inset). (**d**). To estimate the molecular mass of the protein by SAXS, real-space *p*(*r*) distribution and a Guinier plot (inset) were made and *I*(0) and *R*_g_ were determined (Supplementary Table S1). Molecular mass was calculated using the *Q*_R_ method.

FL PAK1 and PAK3 were incubated with a catalytic quantity of CDC42:GTPγS (1:100 CDC42:PAK) and millimolar quantities of adenosine triphosphate (ATP) and magnesium. Auto-phosphorylation was confirmed by denaturing LC–MS; deconvoluted spectra for each protein construct showed multiple 80 kDa additions to the mass of the proteins, indicative of multiple phosphorylation states. After activation, the minimal phosphorylation state of PAK1 had two phosphorylated residues (Figure 3a). The phosphorylated PAK proteins were then purified by SEC to remove excess nucleotide and CDC42, and the oligomeric states determined. Phosphorylated PAK1 protein was almost exclusively monomeric by AUC-SV (Figure 3b).

Using HPLC-SAXS, the molecular mass of phosphorylated PAK1 and PAK3 was then determined (e.g., Figure 3c,d for PAK1) and the results compared with that of the unphosphorylated proteins. SAXS estimates of molecular mass for the phosphorylated protein samples correlated well with that determined for the unphosphorylated proteins (Table 1), indicating that they are monomeric in solution after undergoing phosphorylation and in the absence of GTPase.

### The crystal structure of the PAK3 kinase domain reveals a thiophosphorylated activation loop

**Figure 4.**
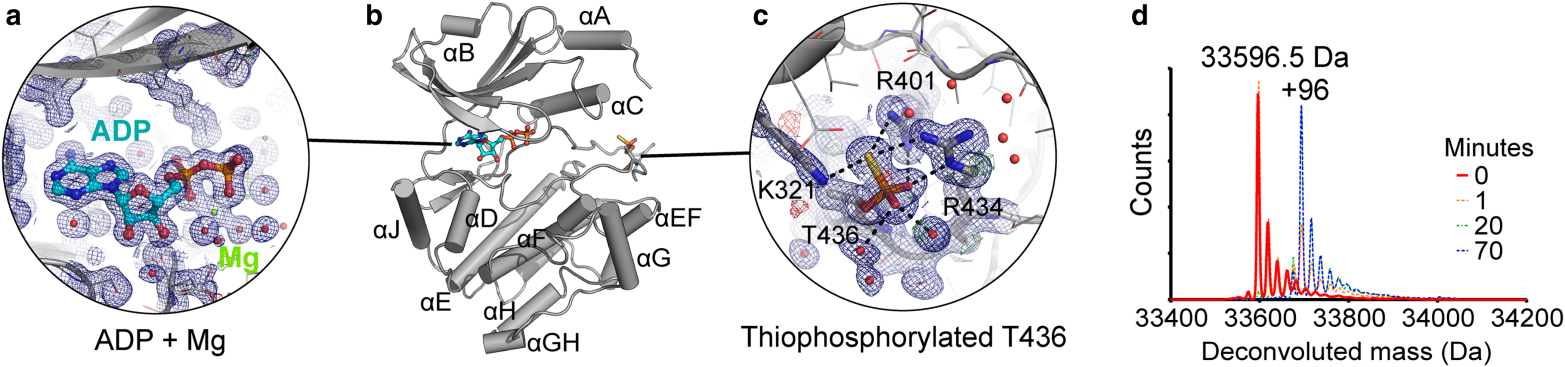
Crystal structure of the PAK3 KD (grey) and key features. (**a**) PAK3 KD was crystallised in the presence of magnesium and ATP-γ-S, which was converted to ADP (cyan). Blue mesh = 2*F*_o_ − *F*_c_ electron density map contoured at 1σ within 4 Å of ADP molecule, green and red mesh = *F*_o_ − *F*_c_ positive and negative difference map contoured at 3σ. Red spheres = water molecules, green spheres = magnesium ions. (**b**) Overview of the crystal structure showing the placement of ADP and the thiophosphorylated activation loop. (**c**) Details of thiophosphorylated residue, T436, including electron density 2*F*_o_ − *F*_c_ map (blue mesh), positive (green mesh) and negative (red mesh) difference density *F*_o_ − *F*_c_ map and key hydrogen-bonding interactions (black dotted lines). (**d**) Overlaid denaturing mass spectra of PAK3 KD incubated in solution with ATP-γ-S and magnesium, taken at various time points. PAK3 rapidly auto-thiophosphorylates using ATP-γ-S, leading to covalent addition of a thiophosphate group (+96 kDa).

To assist modelling the PAK3 FL SAXS data (see below), we solved the crystal structure of PAK3 KD in complex with ATP-γ-S to 1.5 Å resolution (Supplementary Table S3). ATP-γ-S is a commonly used ‘hydrolysis resistant’ ATP analogue, so we were surprised to discover that in the crystal structure ADP and magnesium ions were resolved at the nucleotide-binding pocket (Figure 4a), while the thiophosphate group of ATP-γ-S had been transferred to the PAK3 activation loop (Figure 4b). This is the first example of a crystal structure of any protein containing a thiophosphorylated residue; in spite of the common use of ATP-γ-S in co-crystallisation (over 130 entries in the PDB currently list ATP-γ-S as a ligand). PAK3 is in an active conformation, with thiophosphorylated activation loop at T436. The sulfur atom of the thiophospho-threonine is clearly resolved in the electron density and makes hydrogen-bonding interactions with K321 from the αC-helix of the kinase N-lobe and R401 and R434 of the activation loop, with maximum donor–acceptor distances in the region of 3.1–3.6 Å, compared with donor-acceptor distances of 2.7–3.0 Å for the phosphate oxygen atoms and their interaction partners (Figure 4c). To verify the capability of PAK3 KD to utilise ATP-γ-S as a substrate, we monitored the thiophosphorylation reaction using denaturing mass spectrometry, whereby covalent addition of the thiophosphosphate group to the activation loop can be observed by a corresponding increase in the deconvoluted mass. Thiophosphorylation of PAK3 was detectable after 1 min of incubation in solution and the protein was fully thiophosphorylated after 1 h (Figure 4d).

Superposition of the kinase C-lobe of the PAK3 structure with those of the phosphorylated PAK1_K299R_ structures (PDB 3q52, 3q53) reveals a large shift in the position of the N-lobe. The interaction of the thiophosphorylated residue in PAK3 with the side chain of K321 in the αC-helix is unique to the PAK3 ADP-bound structure. In both the *apo* and ATP-bound phosphorylated PAK1_K299R_ structures, the αC-helix and the phosphate-binding loop are both shifted upwards away from the activation loop by 6–8 Å resulting in a much more open conformation relative to that seen in PAK3. In the PAK3 structure, the salt bridge linking K312 of the β3 strand (equivalent to K299 in PAK1) to E328 of the αC-helix, highly conserved in protein kinases, indicates that the kinase is an active conformation, whereas in the PAK1 structures, this interaction is disrupted by the K299R mutation. The overall fold of the thiophosphorylated PAK3 activation loop has high similarity to the phosphorylated PAK1 activation loop, with minor shift at the main chain around T415-S419 (PAK1 numbering) where crystal packing influences the conformation.

The PAK3 structure also allows mapping of disease-linked mutations (Supplementary Table S4 and Supplementary Figure S4). For example, several mutations in the KD of PAK3 have been linked to non-syndromic X-linked intellectual disability [12,14,64–67] and the exon 10 c.1094C>A mutation leads to a p.A365E substitution (isoform 2 numbering) which would lead to a disruption of the kinase C-lobe fold in the C-terminal region. Another known missense mutation, c.1337G>C in exon 7, leads to p.W446S [66], which again would have a negative impact on the protein fold. A list of the known mutations and potential consequences on PAK3 function are given in Supplementary Table S4, along with any experimental evidence previously reported in the literature.

### Solution structures of full-length group A PAKs reveal an elongated but compact shape

**Figure 5.**
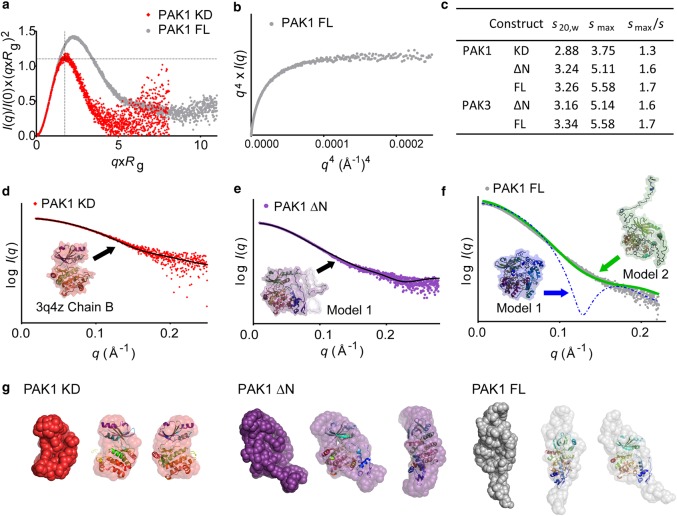
Group A PAKs have an elongated but compact folded shape before activation. (**a**) The dimensionless Kratky plot for PAK1 KD (red) and PAK1 FL (grey). Guinier's approximation for a globular particle is shown at √3 with magnitude 1.04 (grey dotted line). (**b**) Porod-Debye plot for PAK1 FL. (**c**) Semi-quantitative shape determination using AUC-derived sedimentation coefficient; *s*_20,w_ = sedimentation coefficient determined experimentally by AUC-SV, extrapolated to standard conditions using SEDNTERP and *s*_max_ = maximum possible sedimentation coefficient for a smooth unhydrated sphere of mass equal to the mass of the protein calculated, in units of Svedbergs (S). (**d**) Simulated SAXS curve for the high-resolution crystal structure of the unphosphorylated KD of PAK1 (3q4z, chain B), black dotted line, fits the experimental data (red) with χ = 0.98. (**e**) Calculated SAXS curve, black line, for Model 1 of PAK1 ΔN (purple) compared with the experimental data, χ = 1.08. (**f**) Calculated SAXS curves for various models of PAK1 FL compared with the experimental SAXS scattering profile (grey dots). The best- and worst-fitting atomic resolution models generated using I-TASSER [50] are shown in green and blue, respectively. (**g**) Dummy residue models generated for PAK1 from the experimental SAXS data using Gasbor (with solvent removed for clarity), superposed with relevant high-resolution crystal structures (left) PAK1 KD SAXS model (red) superposed with PDB 3q4z chain B (centre). PAK1 ΔN (purple) and (right) FL PAK1 (grey), both superposed with PDB 1f3m chains A and C (residues 70–149 and 248–545).

The SAXS profiles of the PAK FL, ΔN and KD proteins were inspected for indications of particle shape and flexibility. The dimensionless Kratky plot with a peak maximum at √3 with the magnitude of 1.04, obeying Guinier's approximation, indicated that the PAK1 KD is a globular and compact particle (Figure 5a). However, for the unphosphorylated PAK FL and ΔN samples, the shift of the peak maximum in the dimensionless Kratky plot to the right compared with that of the PAK1 KD construct indicates asymmetry (e.g., Figure 5a and Supplementary Figure S5). For all samples, a plateau was seen in the Porod-Debye plot (Figure 5b and Supplementary Figure S5) with a Porod exponent approaching 4 (Supplementary Tables S1 and S2), suggesting that they are generally compact and rigid rather than flexible, extended molecules. It is possible to gain an alternative estimate of the overall shape and globularity of a protein from its sedimentation coefficient (*s*) by determining the ratio of *s*_max_/*s*, with values 1.2–1.3 expected for a compact globular protein, values in the range of 1.5–1.9 indicating asymmetry, and highly elongated proteins have a ratio of *s*_max_/*s* of 2.0–3.0 or greater [46]. Having previously determined *s* for both PAK1 and PAK3 using AUC-SV, a ratio of 1.6–1.7 was calculated for the inactive ΔN and FL, compared with a ratio of 1.3 for the catalytic domain constructs. This is in good agreement with the SAXS data that suggest the catalytic domain is essentially globular, whereas the FL proteins are asymmetric and elongated by comparison.

Using atomic resolution modelling, the crystal structure of unphosphorylated PAK1 KD, with unfolded activation loop (chain B in PDB 3q4z) fit the experimental SAXS data extremely well (χ = 0.98), suggesting that for the isolated catalytic domain, the structure determined crystallographically is representative of that in solution (Figure 5d). For the PAK1 and PAK3 ΔN samples, the best-fitting models showed an extended, asymmetric conformation with a good overall fit to the data and values of χ approaching 1 (Figure 5e, Supplementary Figure S6 and Supplementary Tables S1 and S2). For the FL proteins, models were generated ranging from highly globular, spherical structures (e.g., Model 1, Figure 5f) to elongated and flexible structures with N-terminal regions that extended out into solvent (e.g., Model 2, Figure 5f). Of these models, the calculated scattering curves of those with the greatest asymmetry fit the experimental scattering data best (e.g., Model 2, χ = 3.6). The spherical models, however, fit the experimental data extremely poorly, with χ values exceeding 10 (χ = 10.6 for Model 1) providing further indication that the FL inactive PAKs are asymmetrical and elongated in shape rather than spherical.

Prior to *ab initio* modelling, quantitative assessment of the uniqueness of the shape reconstruction [54] indicated that models generated using the scattering data were likely to be unique (Supplementary Tables S1 and S2). An *ab initio* dummy residue model was then constructed for PAK1 KD, which was in excellent agreement with the high-resolution crystal structures, indicating the quality of the SAXS data (Figure 5g). A similar result was obtained for phosphorylated PAK1 KD (Supplementary Figure S6). *Ab initio* dummy residue models for PAK1 FL and ΔN resulted in elongated and asymmetrical structures with χ in the range of 0.9–1.0 when compared with the experimental data (Supplementary Table S1). Superposition of the crystal structure of the PAK1 KD complexed with the auto-inhibitory fragment (PDB 1f3m chains A and C), indicated that the N-termini of the proteins probably extends into the area below the C-lobe (Figure 5g). In the crystal structure of the AID complexed with the KD (PDB 1f3m), residues 78–86 undergoes dimerisation with the same strand of a second auto-inhibitory fragment from the asymmetric unit, but in solution, we have found the proteins to be monomeric; therefore, it is likely this N-terminal segment occupies a different conformation in solution. Similar data were obtained for PAK3 FL and ΔN samples (see Supplementary Figure S6 and Supplementary Table S2).

The SAXS profile data for the phosphorylated proteins matched closely that of the unphosphorylated proteins, again resulting in dummy atom models that are elongated and asymmetrical (Supplementary Figure S6) and with a Porod exponent of 4 indicating rigidity (Supplementary Figure S5 and Supplementary Tables S1 and S2). It is evident from the SAXS data that there is limited flexibility in the phosphorylated protein; therefore, it appears that the kinase and regulatory segment are connected in a relatively rigid manner and organised as a single, compact entity, rather than acting as two spatially distinct domains hinged by a flexible linker. The SAXS data suggest that the overall envelope of the FL group A PAKs is similar both prior to and following auto-phosphorylation.

## Discussion

**Figure 6.**
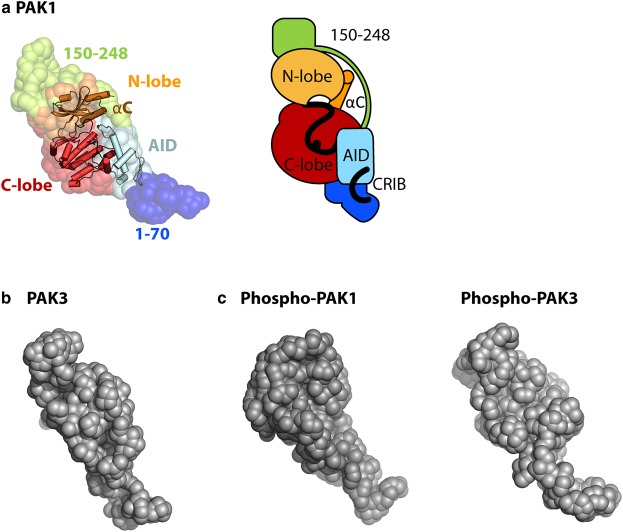
Hypothetical model of group A PAK auto-inhibition. (**a**) PAK1 FL SAXS dummy residue model (spheres, left) superposed with the crystal structure of the PAK1 KD–AID complex (PDB 1f3m) and possible positioning of remaining PAK1 residues. (**b**) SAXS solution structure for PAK3 FL and (**c)** auto-phosphorylated PAK1 and PAK3 FL.

The inability of the FL PAK proteins to form dimers in solution implies that they must be auto-inhibited in *cis*. Combining the SAXS data with the known binding mode of the AID to the KD from the previous crystal structure [22], it is possible to create a model of the auto-inhibited PAK1 FL (Figure 6a). The KD bound to the AID forms the core of the structure, fitting into the central wider portion of the SAXS envelope. The N-terminal 70 residues are likely positioned next to the C-terminal lobe of the KD, as seen from comparison between the PAK1 FL and PAK1 ΔN SAXS reconstructions (Figure 5g), and the region linking the KD and AID (residues 150–248) must, therefore, occupy the remaining end of the SAXS envelope to connect these domains. The PAK3 FL SAXS envelope has a similar shape to that of PAK1 FL including the central bulge that fits the KD (Figure 6b and Supplementary Figure S5), and atomistic models generated for FL PAK3 using the PAK3 KD structure and the AID of PAK1 as a template closely matched the experimental SAXS data (Supplementary Figure S5). These models are in agreement with the *in vitro* kinetic studies of PAK2 that showed that auto-phosphorylation initially occurs in *cis*, followed by amplification of the signal in *trans* [26]. While the SAXS envelopes for the un-activated PAK1 and PAK3 FL show a similar shape, the phosphorylated PAK1 and PAK3 envelopes appear different (Figure 6c). The kinetics of activation [25,26] support a model in which phosphorylation following CDC42 binding causes a rearrangement that prevents *cis*-auto-inhibition. It is currently unknown whether this rearrangement is a small movement of the activation loop of the KD or a larger rearrangement of KD, AID and N-terminal regions.

The previous dimeric model of un-activated PAK1 began with the observation of an asymmetric dimer (an unusual occurrence) in the crystal structure of the PAK1_K299R_ KD (residues 248–545) in complex with a truncated portion of the N-terminal auto-inhibitory segment (residues 70–149) [22]. SEC analysis of PAK1 ΔN (70–545) was consistent with a dimer, as was dynamic light scattering and a SE analysis [22]. Since the observed dimeric interface involved residues 78–86, at the N-terminus of the ΔN construct, one hypothesis is that the presence of PAK1 FL residues 1–69 prevents dimer formation by changing the dynamics of the 78–86 region and/or binding the N-terminus against the KD in a compact arrangement as seen in our SAXS data. However, to test this hypothesis, we also assessed oligomerisation of PAK1 and PAK3 ΔN constructs, which were also monomeric in all of our measurements.

Subsequent published data included a SEC analysis of rat PAK1_L404S_ which showed that this species had a mass somewhere between that expected for monomeric and dimeric species [21], while by overexpression of PAK1_K299R_ in human 293T cells followed by cell lysis, Parrini et al. [61] suggested that, by running the resulting cell lysate over an SEC column, dimer could be detected when comparing the mass of the main component of the cell lysate to two molecular mass markers. However, the difficulties of obtaining accurate estimates of molecular mass using SEC are well known [46,68–71], and retention times are influenced by factors such as particle shape, sphere of hydration and frictional drag. We have shown that molecular mass estimates for PAK1 or PAK3 FL and PAK1 or PAK3 ΔN are consistently overestimated by SEC when compared with molecular mass calibration curves constructed using conventional globular molecular mass standards, but that molecular mass consistent with monomeric rather than dimeric species are obtained when also taking particle shape and surface properties into account (Supplementary Figure S3). This demonstrates the importance of using multiple independent techniques for assignment of oligomeric state.

A further important observation is that previous use of the K299R PAK1 mutant for assessment of oligomerisation may have resulted in experiments using a mixture of phosphorylated and unphosphorylated PAK1. In 2011, a crystal structure of the K299R mutant revealed that it was auto-phosphorylated at T423 on its activation loop (PDB 3q52), and further analysis confirmed that the protein retained basal activity [35]. Given the known transactivation ability of PAK1 [26], it is possible that a mixture of phosphorylated and unphosphorylated PAK1 could dimerise in solution.

Finally, a recent structure of the PAK4:CDC42 complex suggested that the GTPase-bound form of this group B PAK family member is surprisingly globular and has an extended interaction interface, rather than two distinct domains connected by a flexible linker [28]. Our data show that a similar mechanism of CDC42 binding to FL group A PAKs is likely, given the observed rigidity of the FL proteins, and altogether suggests that group A and group B PAKs both form compact monomeric structures.

## Abbreviations

AID: auto-inhibitory domain
AUC-SE: analytical ultracentrifugation–sedimentation equilibrium
AUC-SV: analytical ultracentrifugation–sedimentation velocity
CRIB: CDC42/Rac interactive binding domain
ESI: electrospray ionisation
GBD: GTPase-binding domain
HPLC-SAXS: high-performance liquid chromatography–small angle X-ray scattering
IMAC: immobilised metal affinity chromatography
LC–MS: liquid chromatography–mass spectrometry
PAKs: p21-activated kinases
SEC: size-exclusion chromatography
