# Supplementary material for: Solution structures and biophysical analysis of full-length group A PAKs reveal they are monomeric and auto-inhibited in cis
Source: Biochem J. 2019 Apr 4;476(7):1037–51. doi: 10.1042/BCJ20180867 (PMC6448136; doi:10.1042/BCJ20180867)
Supplement: Supplementary Tables and Figures [file BCJ-476-1037-s1.pdf]

Supplementary Information

Supplementary Table 1. HPLC-SAXS for PAK1.

a) SAMPLE DETAILS	**PAK1 KD**	**PAK1 ΔN**	**PAK1 FL**	**PAK1 FL**	**p-PAK1 KD**	**p-PAK1 FL**
UniProt sequence ID (residues in construct)	Q13153-1 (248-545)	Q13153-1 (70-545)	Q13153-1 (1-545)	Q13153-1 (1-545)	Q13153-1 (248-545)	Q13153-1 (1-545)
Extinction coefficient	29910	41370	42860	42860	29910	42860
Mass from chemical composition for unphosphorylated species (Da)	33441.7	53308.5	60734.1	60734.1	33441.7	60734.1
Phosphorylation state (determined using denaturing LC-MS)	Un-phosphorylate d	Un-phosphorylate d	Un-phosphorylate d	Un-phosphorylate d	phosphorylate d	phosphorylate d
loading concentration (measured by Nanodrop UV-vis spectrometer) (mg/mL)	**8.9**	**15.4**	**15**	**32**	**8.3**	**11.2**
Injection volume (µL)	50	50	50	50	50	50
Flow rate (mL/min)	0.16	0.16	0.16	0.16	0.16	0.16
b) STRUCTURAL PARAMETERS						
Guinier						
I*(0) (cm^{-1})*	1.85E-02	1.10E-02	9.01E-02	1.69E-01	2.12E-02	5.72E-02
R_g*(Å)*	22.70	29.51	37.44	35.67	21.70	33.88
Volume (Å^{-3}) (using I*(0) from Guinier)*	5.77E+04	1.10E+05	1.43E+05	1.39E+05	5.74E+04	1.33E+05
Porod exponent	3.9	3.9	3.9	3.9	3.8	3.9
M *(kDa) (Guinier) determined from* Q_R (1)	32	54	62	66	32	63
P*(r) analysis*						
I*(0) (cm^{-1})*	2.05E-02	1.20E-02	8.62E-02	1.64E-01	2.40E-02	5.72E-02
R_g *(Å)*	21.26	29.83	35.47	35.92	21.61	33.26
d_{max} *(Å)*	68	104	129	138	68	109
Porod volume (Å^{-3}) (using real space I*(0))*	6.30E+04	1.10E+05	1.41E+05	1.31E+05	6.40E+04	1.24E+05
M *(kDa) (real space) determined from* Q_R (1)	42	55	60	62	40	57
c) SHAPE MODEL-FITTING RESULTS						
Ambimeter						
Number of compatible shape categories (out of 14112 total)	16	14	1		8	21
Ambiguity score (<1.5 = potentially unique solution)	1.204	1.146	0		0.9031	1.322
GASBOR (default parameters)						
q *range for fitting (Å^{-1})*	0.0123-0.2920	0.0093-0.2680	0.0114-0.2253		0.0140-0.3497	0.0107-0.2405

X^2	0.76	0.57	0.92		0.66	0.81
d) ATOMISTIC MODELLING						
Template for best model	3q4z:B	1f3m:A,C	2j7t:A		3q52 chain A	
q *range for all modelling* ($Å^{-1}$)	0.0022-0.3229	0.0092-0.3006	0.0085-0.2255			
FoXS (values reported for best model)						
X	0.98	1.08	3.57		0.94	
Predicted R_g *(Å)*	20.14	29.29	30.99		19.73	
c*1,* c*2*	0.99, 4.00	1.05, -0.37	0.99, 2.41		1.00, 4.00	

Supplementary Table 2: HPLC-SAXS for PAK3

a) SAMPLE DETAILS	**PAK3 ΔN**	**PAK3 FL**	**PAK3 FL**	**p-PAK3 FL**
UniProt sequence ID (residues in construct)	O75914-2 (65-544)	O75914-2 (1-544)	O75914-2 (1-544)	O75914-2 (1-544)
Extinction coefficient	42860	42860	42860	42860
Mass from chemical composition for unphosphorylated species (Da)	53871.1	60780	60780	60780
Phosphorylation state (determined using denaturing LC-MS)	unphosphorylated	unphosphorylated	unphosphorylated	phosphorylated
loading concentration (measured by Nanodrop UV-vis spectrometer) (mg/mL)	**5**	**11.3**	**22**	**10.3**
Injection volume (µL)	50	50	50	50
Flow rate (mL/min)	0.16	0.16	0.16	0.16
b) STRUCTURAL PARAMETERS				
Guinier				
I*(0) (cm^{-1})*	3.50E-02	5.983-02	1.27E-01	5.91E-02
R_g *(Å)*	29.79	35.30	35.74	35.6
Volume (Å^{-3}) (using I*(0) from Guinier)*	1.10E+05	1.46E+05	1.44E+04	1.46E+05
Porod exponent	4	3.8	3.9	3.8
M *(kDa) (Guinier) determined from* Q_R (1)	55	69	69	69
P*(r) analysis*				
I*(0) (cm^{-1})*	3.90E-02	6.06E-02	1.24E-01	5.89E-02
R_g *(Å)*	32.03	36.79	36.15	35.13
d_{max} *(Å)*	119	142	139	119
Porod volume (Å^{-3}) (using real space I*(0))*	1.20E+05	1.48E+05	1.41E+04	1.46E+05
M *(kDa) (real space) determined from* Q_R *(1)*	63	68	66	69
c) SHAPE MODEL-FITTING RESULTS				
Ambimeter				
Number of compatible shape categories (out of 14112 total)	28	16		13
Ambiguity score (<1.5 = potentially unique solution)	1.447	1.204		1.114
GASBOR (default parameters)				
q *range for fitting (Å^{-1})*	0.0124-0.2543	0.0052-0.2220		0.0061-0.2277
X^2	0.38	0.8		0.9
d) ATOMISTIC MODELLING				
Template for best model	6f3d:A and 1f3m:A	6f3d:A and 1f3m:A		
q *range for all modelling (Å^{-1})*	0.0023-0.3697	0.0052-0.3696		

FoXS (values reported for best model)		
X	0.73	1.31
Predicted R_g *(Å)*	30.02	35.55
c*1,* c*2*	1.05, 0.25	0.99, 2.47

Supplementary Table 3: X-ray data collection and refinement statistics

**Complex**	**Thiophosphorylated PAK3 (ADP/Mg)**
PDB ID code	6FD3
**Data Collection**	
Space group	$I4_1$
Cell constants:	
a, b, c (Å), α, β, γ (°)	108.8, 108.8 , 57.93, 90, 90, 90
Resolution (Å)[a]	54.41-1.52 (1.56-1.52)
Unique observations[a]	52114 (3802)
Completeness (%)	100.0 (96.0)
Redundancy[a]	6.4 (6.3)
R_{merge}[a]	0.05 (1.55)
R_{pim}[a]	0.02 (0.67)
<I/σ(I)>[a]	16.1 (1.4)
CC½ [a]	1.00 (0.51)
**Refinement**	
Resolution (Å) [a]	1.52-54.41 (1.52-1.55)
MR model	3Q4Z chain A
Copies in ASU	1
R_{work}, R_{free}[a]	0.175, 0.190 (0.331, 0.338)
Number of atoms	2705
Average B factor (Å^2)	28
r.m.s.d. (bonds) (Å)	0.006
r.m.s.d. (angles) (°)	0.956
**Crystallisation conditions**	20% PEG 3350, 0.2 M magnesium formate, 293 K.

[a] Values in parentheses indicate data for the highest resolution shell

ASU, asymmetric unit; r.m.s.d. root mean square deviation.

Supplementary Table 4: Known mutations located in the catalytic domain of PAK3 in non-syndromic X-linked intellectual disability

Mutation in PAK3a (isoform 2)	Equivalent position in PAK3b (isoform 1)	Likely effect on PAK3 function	Experimental evidence for effect on PAK3 function
p.A365E (2)	A380	Destabilising	Reduced stability in cycloheximide assay (3)
p.K389N (3)	K404	Decrease catalytic efficiency and substrate binding	Phosphorylation of MBP impaired *in vitro.* No effect on stability in cycloheximide assay (3)
p.R419* (4)	R434	Truncation, destabilisation and loss of kinase activity	Autophosphorylation and phosphorylation of MBP *in vitro* abolished (4). Reduced stability in cycloheximide assay (3)
p.Y427H (5)	Y442	Destabilising and loss of possible tyrosine phosphorylation site, could interfere with interaction of binding partners and substrates	*In silico* modelling suggests destabilisation (5)
p.W446S (6)	W461	Destabilising	Reduced stability in cycloheximide assay (3)
p.R493C (7)	R508	Stability or interaction with binding partners affected?	

Supplementary Figures

Supplementary Figure 1

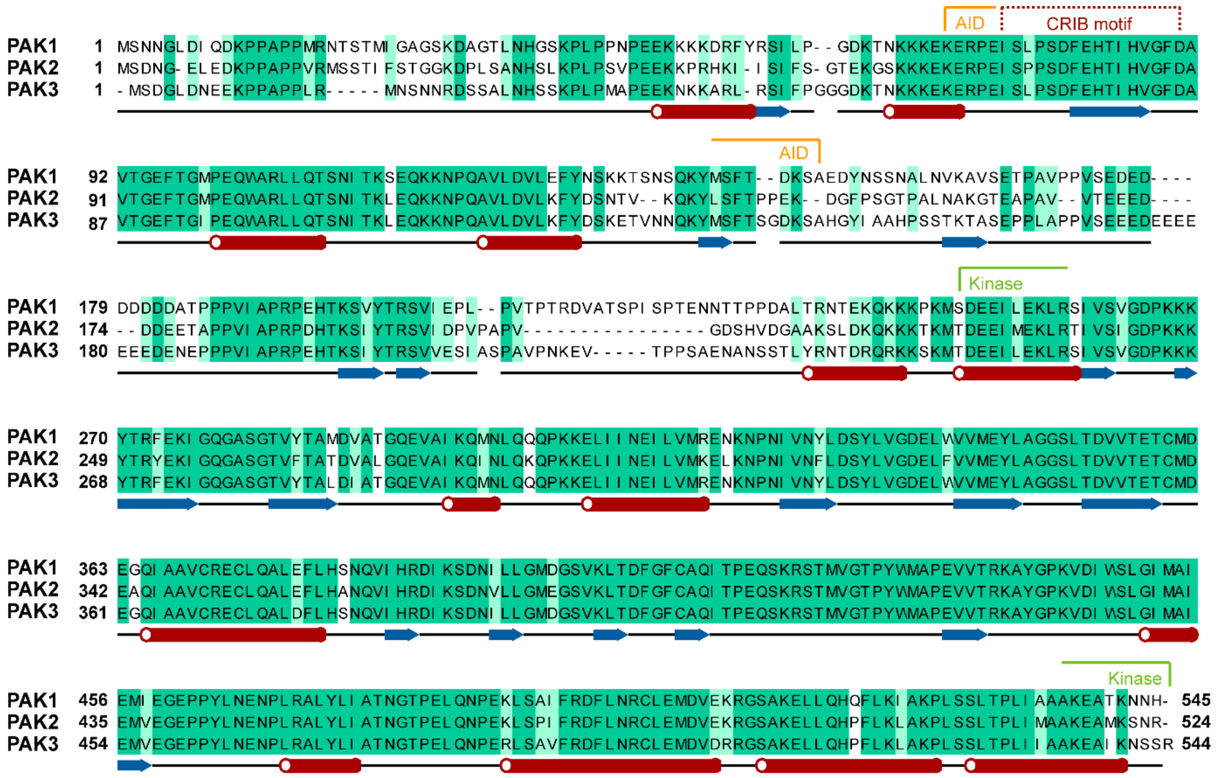

Supplementary Figure 1. Sequence alignment of PAK1, PAK2 and PAK3 where dark green indicates sequence conservation. Secondary structure elements of PAK1 are shown below, where red cylinders indicate alpha helices and blue arrows indicate beta strands. Domain boundaries and interaction motifs are indicated above, where AID = autoinhibitory domain and CRIB = CDC42/Rac-interaction/binding motif.

Supplementary Figure 2

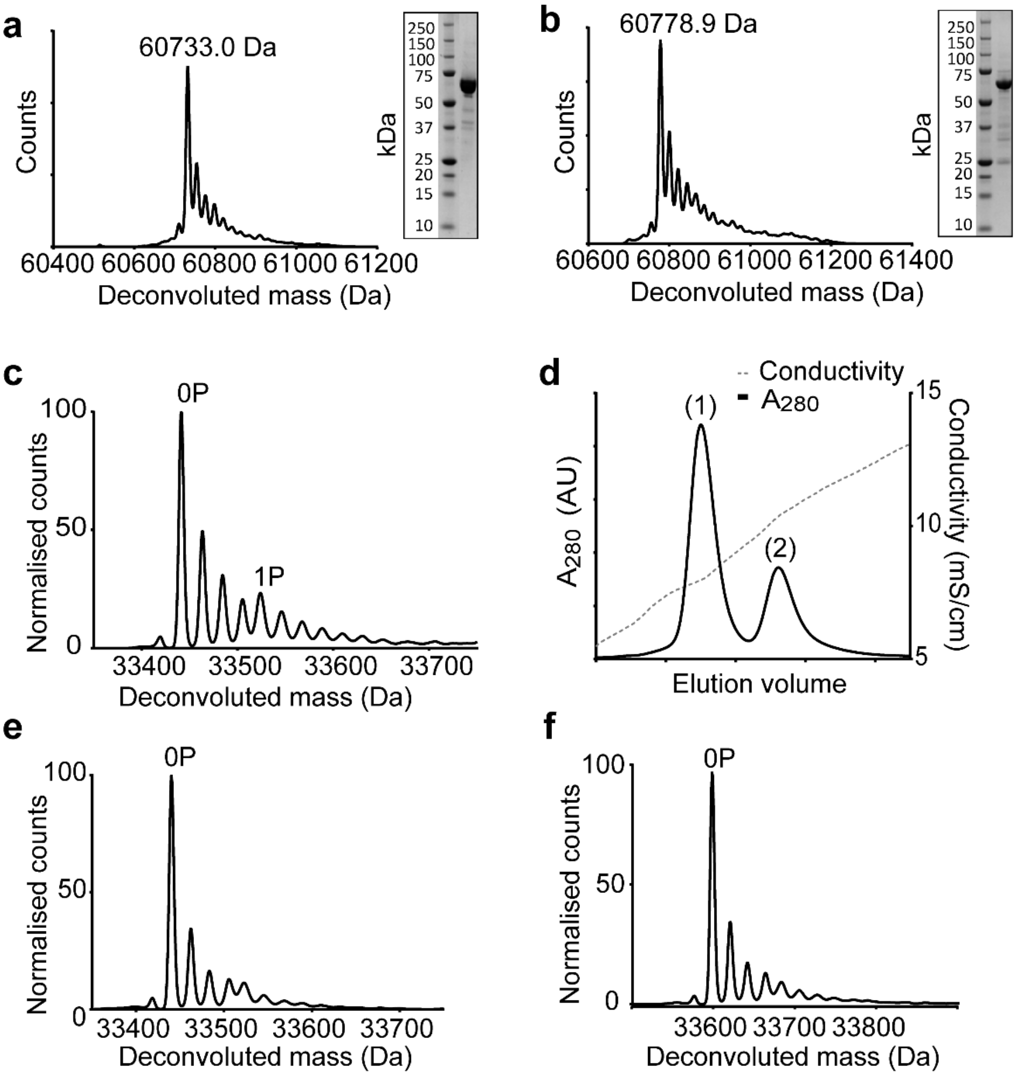

Supplementary Figure 2. Full-length and truncated Group A PAK proteins can be produced in their unphosphorylated (inactive) forms. **a and b.** Deconvoluted denaturing mass spectrum and (inset) SDS-PAGE gel analysis of purified unphosphorylated full-length PAK1 (**a**) and PAK3 (**b**). PAK protein samples shown in lane 2 against molecular weight marker (lane 1). **c.** Deconvoluted mass spectrum of PAK1 KD prior to ion exchange step, with both unphosphorylated (0P) and phosphorylated (1P) material present as indicated. **d.** Representative ion exchange chromatogram of PAK KD samples resolved into two separate peaks, (1) and (2), according to phosphorylation state. **e.** Denaturing LC-MS of PAK1 KD sample from peak (1) following ion exchange showing that the sample consists only of unphosphorylated material. **f.** Denaturing LC-MS of PAK3 KD sample following ion exchange, similar to PAK1 KD sample, showing sample consists of unphosphorylated material only.

Supplementary Figure 3

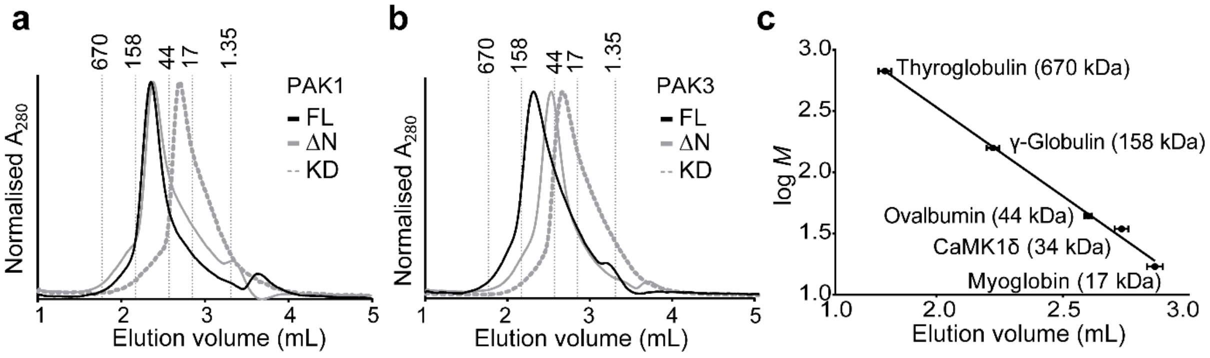

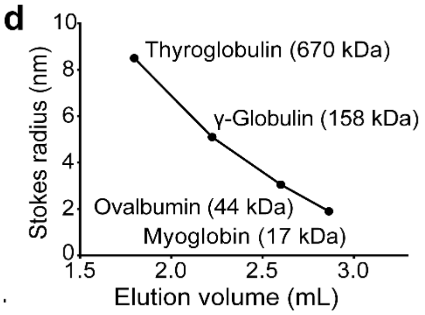

e

	Construct	M_{CALC} (kDa)	M_{SEC1} (kDa)	$s_{20,w}$ (S)	R_s (nm)	M_{SEC2} (kDa)
PAK1	FL	60.7	106.9	3.26	4.5	61.8
	ΔN	53.3	89.7	3.24	4.2	56.7
	KD	33.4	30.0	2.88	2.9	35.3
PAK3	FL	60.8	124.2	3.34	4.7	66.0
	ΔN	53.9	65.6	3.16	3.6	48.0
	KD	33.6	31.6			

Supplementary Figure 3. Size-exclusion chromatography (SEC) derived molecular mass estimates for PAK proteins. **a** and **b.** Representative analytical SEC elution profiles of unphosphorylated recombinant human PAK1 and PAK3 constructs. **c**. Calibration curve of molecular weight compared to SEC elution volume using molecular weight standards. **d**. Calibration curve for calculation of Stokes radius of PAK constructs. **e**. Table of SEC-derived molecular mass estimates for PAK constructs, where M_{CALC} indicates the calculated mass based on the amino acid sequence of the protein. M_{SEC1} is the molecular mass derived using calibration curve shown in c.; $s_{20,w}$ is the experimentally determined sedimentation coefficient converted to standard conditions using SEDNTERP; R_s is the Stokes radius determined using the calibration curve shown in d.; and M_{SEC2} is the molecular mass derived using both the Stokes radius and sedimentation coefficient (as described in Experimental Methods).

Supplementary Figure 4

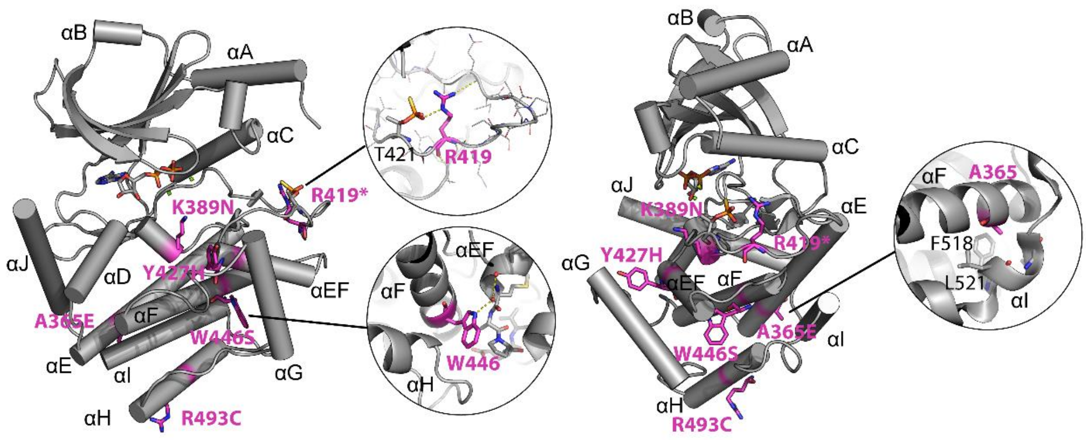

Supplementary Figure 4: Known mutations found in the catalytic domain of PAK3 in non-syndromic X-linked intellectual disability (shown in magenta) mapped onto the PAK3 crystal structure. Three of the sites are highlighted (inset) and polar interactions are indicated (yellow dotted lines). Residues are labelled according to the PAK3a (isoform 2) sequence numbering.

Supplementary Figure 5

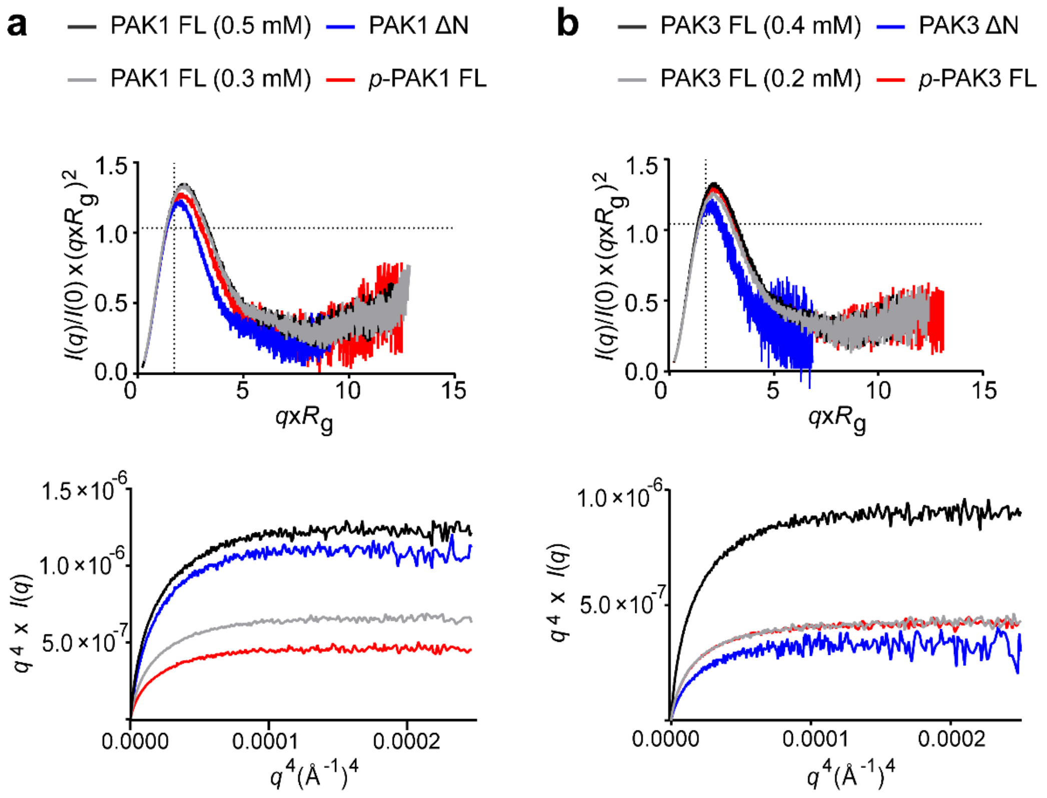

Supplementary Figure 5: HPLC-SAXS normalised Kratky (top) and Porod-Debye (bottom) plots for various PAK1 and PAK3 samples. **a.** Full-length PAK1 (PAK1 FL) at 0.5 mM (black line) and 0.3 mM (grey line); PAK1 ΔN (blue) and phosphorylated PAK1 FL (*p*-PAK1 FL, red). **b.** Full-length PAK3 (PAK3 FL) at 0.5 mM (black line) and 0.3 mM (grey line); PAK3 ΔN (blue) and phosphorylated PAK3 FL (*p*-PAK3 FL, red).

Supplementary Figure 6

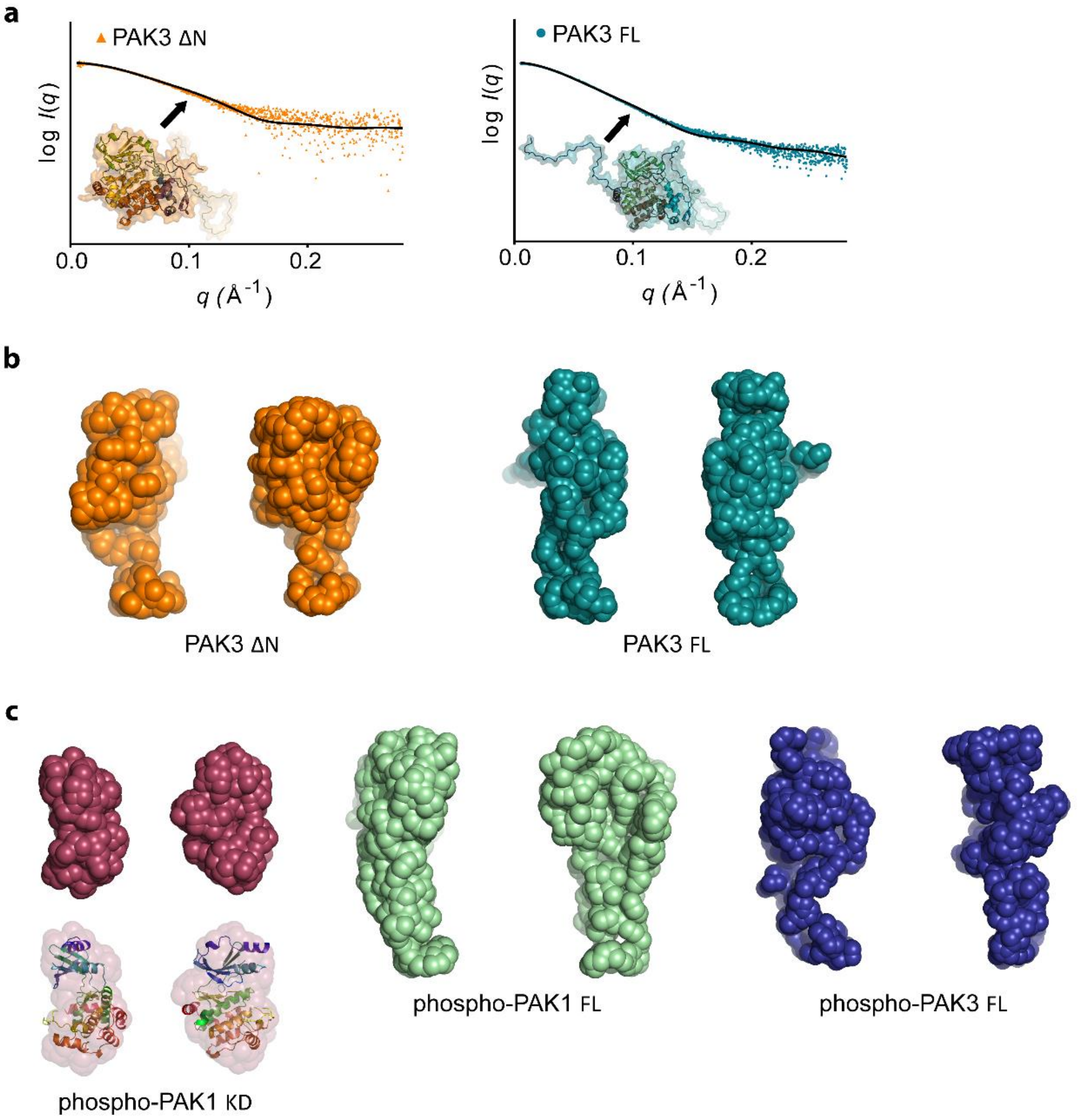

Supplementary Figure 6: Additional SAXS modelling data for PAK1 and PAK3. **a.** Atomic modelling of PAK3 ΔN (orange) and PAK3 FL (teal) unphosphorylated samples using the crystal structure of PAK3 (6fd3) and auto-inhibitory fragment from PAK1 (1f3m Chain A) as a template. **b.** *Ab initio* SAXS models for unphosphorylated PAK3 ΔN (orange) and FL (teal). **c.** *Ab initio* models for PAK1 and PAK3 constructs after auto-phosphorylation: (red) isolated kinase domain (KD) of PAK1 and superposition with the X-ray crystal structure of the phosphorylated PAK1 kinase domain, (green) PAK1 full-length (FL) and (blue) PAK3 FL.

Supplementary References

1. Rambo RP, Tainer JA. Accurate assessment of mass, models and resolution by small-angle scattering. Nature. 2013;496(7446):477-81.
2. Gedeon AK, Nelson J, Gecz J, Mulley JC. X-linked mild non-syndromic mental retardation with neuropsychiatric problems and the missense mutation A365E in PAK3. American journal of medical genetics Part A. 2003;120a(4):509-17.
3. Magini P, Pippucci T, Tsai IC, Coppola S, Stellacci E, Bartoletti-Stella A, et al. A mutation in PAK3 with a dual molecular effect deregulates the RAS/MAPK pathway and drives an X-linked syndromic phenotype. Human molecular genetics. 2014;23(13):3607-17.
4. Allen KM, Gleeson JG, Bagrodia S, Partington MW, MacMillan JC, Cerione RA, et al. PAK3 mutation in nonsyndromic X-linked mental retardation. Nature genetics. 1998;20(1):25-30.
5. Hertecant J, Komara M, Nagi A, Al-Zaabi O, Fathallah W, Cui H, et al. A de novo mutation in the X-linked PAK3 gene is the underlying cause of intellectual disability and macrocephaly in monozygotic twins. European journal of medical genetics. 2017;60(4):212-6.
6. Peippo M, Koivisto AM, Sarkamo T, Sipponen M, von Koskull H, Ylisaukko-oja T, et al. PAK3 related mental disability: further characterization of the phenotype. American journal of medical genetics Part A. 2007;143a(20):2406-16.
7. McMichael G, Bainbridge MN, Haan E, Corbett M, Gardner A, Thompson S, et al. Whole-exome sequencing points to considerable genetic heterogeneity of cerebral palsy. Molecular psychiatry. 2015;20(2):176-82.
